# Toward genomic personalization of breast cancer radiotherapy: foundations, challenges, and a roadmap for clinical integration

**DOI:** 10.1016/j.breast.2026.104733

**Published:** 2026-02-11

**Authors:** Pierre Loap, Irene Buvat, Gilles Crehange, Youlia Kirova

**Affiliations:** a Department of Radiation Oncology, Institut Curie, Paris, France; b Advanced Therapy in Radiation Oncology and Molecular Imaging Characterisation (ATOMIC), Institut Curie, Orsay, France; c Proton Therapy Center (CPO), Institut Curie, Orsay, France

**Keywords:** Breast cancer, Radiogenomics, Radiosensitivity index (RSI), Genomic-adjusted radiation dose (GARD), Personalized radiotherapy

## Abstract

Personalizing radiotherapy dose in breast cancer remains a major unmet need, as current treatment paradigms rely on uniform prescriptions that overlook interpatient variability in intrinsic radiosensitivity. Over the past decade, transcriptome-based biomarkers such as the Radiosensitivity Index (RSI) and its radiobiological extension, the Genomic-Adjusted Radiation Dose (GARD), have emerged as promising tools capable of quantifying this biological heterogeneity and linking it to expected therapeutic effectiveness. Retrospective clinical studies across diverse breast cancer cohorts have consistently demonstrated that RSI and GARD correlate with locoregional control, identify radioresistant subgroups that may benefit from dose escalation, and reveal radiosensitive tumors for which de-escalation may be safely explored. These findings challenge the assumption that radiation response is uniform within histological or molecular subtypes and highlight the opportunity for biologically tailored dosing. Yet despite early evidence, translation into clinical practice remains limited. Key barriers include the absence of prospective validation, heterogeneous analytic pipelines for RNA sequencing and RSI computation, uncertainty regarding optimal biomarker timing in the neoadjuvant era, and sensitivity of bulk transcriptomic assays to spatial and microenvironmental heterogeneity. Addressing these challenges will require standardization, consensus on clinically meaningful GARD thresholds, and coordinated international efforts to define methodological and regulatory pathways. Emerging approaches in radiomics, digital pathology, and multimodal artificial intelligence may further refine radiosensitivity assessment and reduce reliance on invasive sampling. As the field progresses, genomic personalization of radiotherapy has the potential to transform breast cancer management by replacing one-size-fits-all prescriptions with biologically informed dose adaptation aimed at maximizing tumor control while minimizing toxicity.

## Highlights

• Genomic signatures reveal substantial heterogeneity in breast radiosensitivity.
• RSI/GARD provide a framework for biology-driven radiotherapy dose adaptation.
• Spatial and temporal variability remain major barriers to biomarker accuracy.
• Standardized analytic pipelines and unified GARD thresholds are essential.
• Safe implementation requires prospective trials and a robust regulatory pathway.

## 1 Introduction

Adjuvant radiotherapy is a central component of breast-conserving treatment [1,2]. It sterilizes residual microscopic disease within the breast [3] and addresses occult tumor deposits in regional lymph nodes, particularly in node-positive patients. Through these mechanisms, adjuvant radiotherapy substantially reduces the risk of locoregional recurrence. Clinical dose prescriptions remain largely standardized. Hypofractionated regimens such as 40.05 Gy in 15 fractions over 3 weeks and ultra-hypofractionated schedules such as 26 Gy in 5 fractions over 1 week are now widely used [[4], [5], [6]], yet are applied uniformly across patients without explicit consideration of tumor biology.

This population-based approach to dosing has two main limitations. The first relates to normal tissue toxicity. Even though modern techniques have markedly reduced radiation exposure to critical organs, residual toxicity risk persists. Cardiac morbidity is of particular concern [7,8], and the strongest predictors of late cardiac events are the mean heart dose and the dose delivered to individual cardiac substructures [9,10]. In current practice, efforts to reduce late effects in higher-risk patients, such as younger individuals or those harboring radiosensitizing germline mutations, rely predominantly on technical optimization of radiation delivery [11,12] rather than on biologically individualized dose modulation.

The second limitation concerns local control. Inter-patient variability in intrinsic tumor radiosensitivity is considerable [[13], [14], [15]], yet remains clinically unmeasured. Patients with residual disease after neoadjuvant systemic therapy exemplify subgroups at high risk of locoregional recurrence [16], but radiotherapy dose remains usually fixed regardless of this elevated risk. Because radioresistance cannot be routinely assessed, standard prescriptions are applied uniformly, and selective dose escalation for biologically radioresistant tumors is not implemented.

These limitations underscore the need for biologically informed dose personalization. Ideally, radiation dose would be adapted to tumor biology, with de-escalation for highly radiosensitive tumors and escalation for resistant disease. Over the last decade, transcriptome-based prognostic signatures derived from large patient cohorts have been developed to associate gene expression patterns with clinical outcomes and, in most cases, to estimate whether adjuvant radiotherapy is necessary or not [[17], [18], [19], [20]]. However, these signatures were not designed to inform dose prescription and therefore cannot support genuine dose individualization.

To address this gap, *in vitro*–derived genomic signatures of radiosensitivity have been proposed to link tumor genomic characteristics with intrinsic radioresponse and to infer the expected biological effectiveness of a given radiation dose. These tools are specifically intended as foundations for biology-guided dose prescription. Nevertheless, they remain at an early developmental stage, and their clinical translation raises critical methodological and safety questions.

A structured translational framework is therefore essential to enable safe early-phase evaluation and to avoid inappropriate under- or overtreatment when integrating genomic radiosensitivity profiling into radiotherapy planning. The aim of this review is to propose such a roadmap, outlining the steps required for rigorous translation of genomics-based assessment of tumor radiosensitivity into clinical practice in breast cancer radiotherapy, from preclinical development through prospective trials and implementation.

## 2 Genomic radiosensitivity signatures in breast oncology

The initial attempts to connect tumor genomics with intrinsic radiosensitivity were grounded in *in vitro* clonogenic survival assays, using the surviving fraction at 2 Gy (SF_2_) as a quantitative proxy of cellular radioresponse. In this framework, baseline gene expression profiles of cancer cell lines are paired with SF_2_, and statistical models are trained to predict radiosensitivity from transcriptomic fingerprints. Three major signatures have emerged from this approach and now occupy a central place in breast oncology translational research: the Radiosensitivity Index (RSI) [21], a 31-gene radiation sensitivity signature (RSS) developed by Kim et al. [22], and a breast-specific RSS proposed by Speers et al. [23]. Each signature refines, in its own way, the training set and learning strategy, evolving from pan-cancer panels to breast-restricted models and from simple regression toward more complex machine learning.

### 2.1 The Linear–Quadratic model as a bridge between SF_2_ and dose individualization

Within the classical linear–quadratic (LQ) model, the radiosensitivity of a cell population is described by two parameters, α and β, according to:

$$\text{SF}\left(d\right)=e^{-\left(\alpha \times d+\beta \times d^{2}\right)}$$

where SF(d) is the surviving fraction after a dose d, α (Gy^−1^) captures the probability that a single radiation track is sufficient to inactivate a cell (a one-hit lethal event), and β (Gy^−2^) quantifies cell death resulting from the interaction of two sublethal lesions separated in time.

The parameter α reflects the burden of immediate, poorly reparable DNA damage, such as double-strand breaks and clustered lesions. It is strongly modulated by intrinsic tumor biology, including cell-cycle distribution, DNA repair capacity, hypoxia, and key mutations such as *BRCA1/2* and *TP53* [24]. As a result, α exhibits wide inter- and intra-tumor variability [25,26], making it a particularly attractive target for biologically driven personalization of dose.

In contrast, β primarily reflects the dynamics of DNA damage signaling and repair and the time window during which sublethal lesions can interact to form irreparable damage. For a given histologic subtype, β tends to show much less inter-patient dispersion than α [25,26]. Under this assumption, differences in SF_2_ across tumors can be attributed predominantly to differences in α.

Rearranging the LQ expression at d = 2 Gy yields:

$$\alpha =-\frac{\ln\;{\text{SF}}_{2}}{2}-2\times \beta$$

If β is held fixed, any SF_2_ value—whether measured experimentally or inferred from a genomic signature—can be converted into a tumor-specific α and, by extension, into an iso-effective biologically effective dose (BED) for a given treatment schedule. Because β is kept constant, such adjustments remain compatible with existing planning systems and standard fractionation schemas, while embedding genuine inter-patient radiosensitivity differences through the SF_2_-derived α.

Genomic signatures that reliably estimate SF_2_ thus supply the missing link between static, population-based protocols and truly quantitative, biology-driven dose prescriptions. They provide a principled route to translating transcriptomic fingerprints into individualized α estimates and, ultimately, into personalized BED targets.

### 2.2 SF_2_-linked gene expression signatures for breast cancer

#### 2.2.1 The Radiosensitivity Index (RSI)

Eschrich et al. developed the Radiosensitivity Index (RSI) [21] by modeling SF_2_ across 48 carcinoma cell lines from the NCI-60 panel. Starting from 7168 probes on the Affymetrix HU-6800 microarray platform, they applied a systems biology network analysis to identify ten hub genes (*AR, c-JUN, STAT1, PKC, RELA, c-ABL, SUMO1, CDK1, HDAC1, IRF1*) that capture central pathways in the radiation response. Gene expression values for these hubs were rank-ordered within each sample, and a linear regression model was trained to predict SF_2_ from these ranks. The resulting fixed linear combination defines a continuous RSI score, with higher RSI values reflecting more radioresistant phenotypes.

Two design choices are particularly important for later clinical translation. First, the regression coefficients were locked at the time of model training and were not re-estimated in subsequent cohorts, ensuring that RSI functions as a predefined biomarker rather than a flexible model. Second, because the algorithm uses rank-normalized expression rather than absolute intensities, it is inherently platform-agnostic, facilitating application across different microarray platforms and, eventually, in both fresh-frozen and formalin-fixed paraffin-embedded (FFPE) specimens once appropriate probe mappings are standardized. Although breast cancer cell lines represent only a minority of the NCI-60 panel, RSI was rapidly applied to breast tumor datasets due to data availability [27] and has become the de facto archetype of a pan-cancer radiosensitivity signature [28].

#### 2.2.2 A 31-gene radiosensitivity signature (Kim et al.)

Kim et al. [22] extended the SF_2_–genomics paradigm by reanalyzing the full NCI-60 cell line panel profiled across four independent platforms (cDNA arrays, HU-6800, U95, U133). SF_2_ values were curated from the literature and correlated with gene expression using Significance Analysis of Microarrays algorithm with a false discovery rate threshold of 10%. This approach yielded 31 genes consistently associated with radiosensitivity across all four platforms and 179 genes reproduced in at least three.

Instead of fitting a single global regression model, the authors combined gene-level correlation metrics with gene set and pathway analyses. Functional enrichment using KEGG and systems biology tools showed that many of the 31 signature genes encode adhesion and cytoskeleton-related proteins such as ITGB5, ACTN1, RAB13, PFN2 and CCND1, embedded in integrin, focal adhesion and actin–cytoskeleton pathways. Integrative network analyses demonstrated extensive cross-talk between these integrin-centered pathways and major oncogenic signaling cascades, including MAPK, PI3K, Wnt and p53, positioning adhesion-related molecules as central mediators of radiosensitivity.

Although breast cancer cell lines constitute only a fraction of the NCI-60 panel, this integrin-centered biology is highly consistent with existing data on breast tumor–stroma interactions. The 31-gene RSS is therefore formally a pan-cancer signature, but its biological plausibility and methodological robustness make it a compelling candidate for breast-specific validation and refinement.

#### 2.2.3 A breast cancer–specific radiosensitivity signature (Speers et al.)

Speers et al. [23] sought to develop a breast-focused radiosensitivity signature by restricting their discovery set to 16 human breast cancer cell lines spanning all major intrinsic subtypes, including triple negative, luminal A, luminal B, and HER2-amplified tumors. SF_2_ values ranged from 17% (highly radiosensitive) to 77% (markedly radioresistant) and, importantly, showed no association with intrinsic subtype. This observation underscored the inadequacy of phenotype-based classification alone for predicting radioresponse and highlighted the need for molecular classifiers.

Using Spearman correlation between SF_2_ and baseline transcriptomic profiles, the authors identified 147 candidate genes. Functional relevance was then tested experimentally: siRNA-mediated knockdown of top-ranked candidates (including TACC1, RND3 and DTL) in the radioresistant MDA-MB-231 cell line resulted in radiosensitization, with enhancement ratios between approximately 1.2 and 1.4, thereby supporting a causal role in modulating radiation response.

To translate these findings into a clinically relevant tool, a Random Forest classifier was trained using clinical data from 343 early-stage breast cancer patients treated with breast-conserving surgery and adjuvant radiotherapy. The endpoint was locoregional recurrence rather than SF_2_, allowing the model to be optimized directly on a clinically meaningful outcome. Through iterative feature reduction, the authors derived a 51-gene breast-specific RSS. Despite this clinical recalibration, the final signature remained strongly enriched for genes involved in cell-cycle checkpoints, ATM signaling and homologous recombination repair, indicating that the core radiosensitivity biology captured *in vitro* was largely preserved.

Receiver operating characteristic analysis demonstrated excellent discrimination in the training cohort and good performance in cross-validation, with area under the curve values around 0.66. Subsequent independent validation cohorts confirmed the prognostic value of the signature for locoregional recurrence and overall survival. Thresholds and cut-offs were predefined and then maintained fixed across analyses, in accordance with standard biomarker development practice.

Table 1 compares the methodological foundations, biological axes and clinical maturity of these three major transcriptomic signatures.

**Table 1:** Comparative features of genomic SF2-radiosensitivity signatures applicable for breast oncology.

Characteristic	RSI (Eschrich et al.)	31-gene RSS (Kim et al.)	Breast-specific RSS (Speers et al.)
**Training dataset**	48 NCI-60 carcinoma cell lines (≈6 breast lines)	Full NCI-60 on four microarray platforms	16 breast cancer cell lines (all intrinsic subtypes)
**Radiosensitivity phenotype**	Clonogenic SF_2_	Literature-derived SF_2_	Clonogenic SF_2_ and clinical locoregional recurrence
**Transcriptomic profiling**	Affymetrix HU-6800; later mapped to U133	Multi-platform: cDNA, HU-6800, U95, U133	Affymetrix Gene ST 1.0; FFPE/RNA-seq-compatible pipeline
**Modeling strategy**	Fixed-coefficient linear model on rank-normalized expression	Consensus 31-gene set; pathway-based approach	Random Forest classifier trained on recurrence outcomes
**Output format**	Continuous RSI score	Continuous z-score (requires calibration)	Binary classification (RSS-High vs RSS-Low)
**Dominant biological axes**	Cell cycle, NF-κB/STAT1, chromatin remodeling	Integrins, focal adhesion, cytoskeleton dynamics	DNA repair, ATM/BRCA1 signaling, G2/M checkpoint
**Functional validation**	None in breast lines	None reported	siRNA knockdown demonstrating radiosensitization
**FFPE/clinical pipeline maturity**	High (rank-based, coefficients locked)	Moderate (calibration required)	High (locked pipeline and cut-offs)

### 2.3 Convergent design principles and mechanistic themes

Although the RSI [21], the 31-gene RSS [22] and the breast-specific RSS [23] arise from different datasets and modeling strategies, they share a common conceptual framework. All three pair genome-wide expression profiling with a quantitative SF_2_ read-out and all retain sufficient structural definition to permit translation into clinical cohorts. RSI is a fixed-coefficient linear score explicitly optimized for portability across tumor types and platforms. The 31-gene RSS is a consensus gene set designed to maximize biological interpretability and platform robustness, rather than to serve as a standalone predictive score. The breast-specific RSS is a breast-restricted, outcome-optimized machine learning model trained directly on locoregional control data.

Despite these differences, several mechanistic motifs recur across signatures. Down-regulation of integrin and adhesion-related genes and reduced cytoskeletal tension are consistently associated with increased radiosensitivity, whereas up-regulation of DNA damage checkpoint and cell-cycle progression genes correlates with radioresistance. These convergent patterns, observed first in heterogeneous cell line panels and then in breast-focused analyses [23], provide a coherent biological rationale for using transcriptomic fingerprints as surrogates of intrinsic radiosensitivity.

When considered together, the preclinical and retrospective evidence indicates that SF_2_-linked gene expression signatures can quantitatively mirror breast tumor radiosensitivity. This body of work constitutes the early phase foundations for translational programs aimed at testing whether such genomic fingerprints—and their derived α estimates—can safely guide individualized radiation dosing in prospective clinical trials.

## 3 Retrospective clinical evidence for RSI and GARD in breast cancer

### 3.1 Radiogenomic modeling and clinical associations in breast cancer

The transition from preclinical models to clinical application requires demonstrating that genomic estimates of radiosensitivity retain predictive value in patient tumors. Across retrospective datasets spanning multiple breast cancer subtypes and treatment contexts, the Radiosensitivity Index and its derivative, the Genomic-Adjusted Radiation Dose (GARD), have shown consistent associations with locoregional control, survival outcomes, and biological features known to modulate radioresponse.

Retrospective analyses of RSI in breast cancer revealed a broad, continuous distribution of radiosensitivity across patient tumors, mirroring the heterogeneity observed in cell lines. This diversity was independent of intrinsic subtype, grade, hormone receptor status, and HER2 expression, indicating that classical clinical phenotyping does not capture the variance in intrinsic radiosensitivity [15]. These findings underscore the potential of transcriptomic biomarkers to add biological resolution that complements, rather than overlaps with, traditional pathological classification.

An advance came with the development of GARD, which integrates RSI-derived SF_2_ into the LQ formalism to express a patient-specific estimate of the biological effect of a given radiation schedule [28,29]. The linear-quadratic model quantifies radiobiological effect through the fraction of viable tumor cells remaining after n fractions of dose d:

n×d×(α+β×d).

The GARD metric captures this biological effectiveness by incorporating a patient-specific α derived from the RSI, assuming β = 0.05 Gy^-2^ uniformly across patients.

$$\text{GARD}=n\times d\times \left(-\frac{\ln\text{RSI}}{2}-0,1+0.05\times d\right)\text{.}$$

In pooled pan-cancer analyses, including substantial subsets of breast tumors, GARD demonstrated strong associations with survival and recurrence outcomes, while total physical dose alone had limited predictive value [29]. These analyses also suggested that a threshold GARD value may exist above which gains in tumor control become clinically meaningful, although this remains to be validated in breast-specific prospective settings.

Within breast cancer specifically, the retrospective application of RSI and GARD has yielded several important observations. In hormone-receptor–positive disease, lower RSI and higher GARD correlate with improved locoregional control following adjuvant radiotherapy [27]. In triple-negative breast cancer (TNBC), where radiotherapy decisions (such as boost delivery) are often influenced by high baseline recurrence risk, RSI displays a particularly broad range [15,30]. This variability suggests that TNBC is not uniformly radioresistant and that a significant proportion of patients may harbor tumors intrinsically more radiosensitive than generally assumed. This heterogeneity provides a compelling rationale for biologically personalized dosing in this aggressive subtype [15].

Published RSI/GARD performance metrics in breast cancer demonstrate statistically significant but heterogeneous associations with locoregional outcomes. Torres-Roca et al. [27] reported that in ER-negative disease (n = 343), RSI-sensitive/intermediate tumors showed substantially lower local recurrence risk versus RSI-resistant (HR 0.33, 95% CI 0.16-0.70, p = 0.002). Within TNBC (n = 126), this association remained significant (HR 0.37, 95% CI 0.15-0.92, p = 0.02). Dose-response analysis demonstrated that luminal RSI-resistant tumors benefited from escalation above 66 Gy (HR 0.23, 95% CI 0.05-0.98, p = 0.03), whereas RSI-sensitive/intermediate patients showed no benefit (HR 0.78, p = 0.42), providing preliminary predictive evidence. Ahmed et al. [30] evaluated GARD in two independent TNBC cohorts. An European cohort (n = 58) demonstrated that GARD≥23.2 versus <23.2 associated with local control (HR 2.5, 95% CI 1.0-7.1, p = 0.05; 5-year rates 79% vs 55%, p = 0.03). The Moffitt validation cohort (n = 55) showed stronger association using GARD≥21 threshold (HR 4.4, 95% CI 1.1-29.5, p = 0.04; 5-year rates 96% vs 71%, p = 0.04).

A critical distinction must be drawn between prognostic and predictive biomarkers. Prognostic biomarkers correlate with outcomes regardless of treatment received, whereas predictive biomarkers identify differential treatment benefit through significant biomarker-by-treatment interaction effects. Scott et al.'s pan-cancer pooled analysis provides the strongest evidence to date, demonstrating significant GARD × radiotherapy interaction for overall survival (p = 0.011) [29], with control analyses showing no association in non-irradiated patients. However, breast-specific interaction testing in adequately powered cohorts remains lacking. Based on current evidence, RSI/GARD should be considered prognostic biomarkers with emerging predictive signals requiring prospective randomized validation demonstrating that GARD-guided dose modification improves outcomes compared to standard approaches.

### 3.2 Breast-specific modifiers and implications for personalized radiotherapy

Further biological insights emerged from studies investigating tumor purity and immune infiltration. Transcriptomic deconvolution analyses showed that breast RSI is sensitive to variations in non-tumor cell content [15]. Tumors with high immune infiltration tend to exhibit lower RSI values [31], likely reflecting the radiosensitivity of infiltrating lymphocytes rather than cancer cells themselves. This phenomenon has implications for both biomarker interpretation and therapeutic strategy, as it suggests that tumor microenvironmental context influences the genomic signal of radiosensitivity. These findings reinforce the need for future clinical studies to incorporate tumor purity correction or refined computational deconvolution approaches.

A significant step was a neoadjuvant breast cancer study which assessed how systemic therapy modifies intrinsic radiosensitivity [32]. Patients with TNBC underwent RNA-sequencing at diagnosis and again on the surgical specimen. GARD values derived from pre-treatment biopsies correlated poorly with those obtained after neoadjuvant therapy, demonstrating that radiosensitivity is not static. Partial responders frequently exhibited an increase in RSI, indicating a shift toward radioresistance, whereas complete responders had no surgical specimen available for post-treatment profiling. The observed RSI increase among partial responders was associated with reduced immune infiltration, suggesting that systemic therapy reshapes the tumor–immune ecosystem in ways that may reduce responsiveness to radiotherapy. These findings raise critical questions regarding the optimal timing of RSI measurement for clinical decision-making and pose a dilemma for patients achieving pathological complete response, for whom no post-treatment tissue exists.

Parallel technical progress has supported clinical translation by enabling RSI computation on formalin-fixed paraffin-embedded specimens through RNA-sequencing [15,31,33], overcoming the limitations of legacy microarray platforms. Standardized computational pipelines now facilitate the derivation of rank-normalized expression required for RSI, while ensuring reproducibility across laboratories. These methodological advances provide the technical foundation for future prospective studies.

Taken together, the retrospective data support the premise that RSI and GARD capture biologically meaningful heterogeneity in breast tumor radiosensitivity. They also highlight the importance of controlling for tumor purity, the need to define the optimal timepoint for biomarker assessment, and the requirement for rigorous validation of clinically actionable GARD thresholds before dose personalization can be implemented safely.

## 4 Prospective translation, dose individualization, and implementation considerations

### 4.1 Sequential clinical development pathway: from early validation to population implementation

With sufficient preclinical and retrospective evidence, the translational challenge shifts toward establishing safe and methodologically robust prospective applications. The central objective is to determine whether adjusting dose according to genomic radiosensitivity improves locoregional control without introducing unacceptable toxicity or loosing tumor control. This requires a structured sequence of clinical investigations spanning early-phase feasibility to late-phase pragmatic implementation.

The initial prospective studies should focus on demonstrating analytic validity, operational feasibility, and biological plausibility. These early trials must verify that RSI and GARD can be reliably obtained from routine clinical specimens, that turnaround times are compatible with radiotherapy workflows [34], and that biomarker variability is within expected bounds. They should also confirm that calculated GARD values correlate with tumor control and prospectively identify candidate GARD thresholds. Importantly, early-phase trials should not modify radiotherapy dose, as safety cannot yet be assured in the absence of validated thresholds.

Once analytic feasibility and biological coherence are established, intermediate-phase (Phase II) trials can begin exploring biomarker-guided dose adaptation in carefully defined patient groups. These studies must be conducted under strict safety constraints: dose escalation should be limited to ranges supported by historical data [15,35], and dose de-escalation should be approached conservatively to avoid undertreatment of potentially radioresistant tumors. In this stage, the goal is not to demonstrate definitive clinical benefit but to evaluate tolerability of genomic-guided dose escalation, early efficacy signals, and the predictive performance of candidate GARD thresholds.

A central challenge for dose-escalation studies is the risk of late toxicity, particularly fibrosis [36]. Breast tissues exhibit notable inter-patient variability in radiation sensitivity, and excessive biologically effective dose can induce irreversible fibrotic changes that compromise cosmetic outcome and quality of life. For this reason, integrating normal-tissue radiosensitivity biomarkers such as the Radiation-Induced Lymphocyte Apoptosis (RILA) assay is highly relevant [37]. RILA provides an estimate of an individual's susceptibility to late fibrosis: patients with low RILA values have a significantly higher risk of severe late effects. Incorporating RILA into dose escalation protocols could help identify patients more likely to tolerate higher GARD-targeted doses, thereby improving safety without undermining the scientific aims of dose personalization.

The ongoing NCT05528133 trial represents an important initial step toward prospective evaluation, though its design reveals the current nascent state of RSI/GARD clinical translation. This randomized study compares standard-of-care whole breast radiotherapy with tumor bed boost versus an approach where the boost is selectively omitted based on RSI classification. However, this trial does not yet implement true individualized dose personalization. It merely uses RSI as a binary decision tool for boost omission versus administration. Patients in both arms receive identical whole breast doses of either 50 Gy in 25 fractions or 42.56 Gy in 16 fractions, with the experimental arm potentially omitting the standard 10 Gy in 5 fraction boost based on favorable RSI. This represents a preliminary feasibility study rather than genuine RSI-guided dose escalation or de-escalation based on predicted radiosensitivity.

As evidence matures, Phase III randomized trials become essential. These studies must test fixed, pre-specified biomarker thresholds derived from retrospective and Phase II data. They should randomize patients to standard-of-care radiotherapy versus GARD-guided dose adjustment, with locoregional recurrence as the primary endpoint and toxicity as a co-primary or major secondary endpoint. Adaptive statistical designs may be particularly advantageous, allowing dynamic refinement of GARD thresholds or stratification rules as accumulating data clarify biomarker–outcome relationships. Importantly, these trials must ensure equity and safety across molecular subtypes, especially in TNBC and post-neoadjuvant settings where radiosensitivity dynamics are most complex [15,32].

Finally, Phase IV implementation requires navigating regulatory, infrastructural, and health-economic considerations. Biomarkers intended to guide therapeutic dose carry stringent regulatory obligations and will likely require certification with harmonized laboratory workflows. Health-economic analyses will be indispensable for demonstrating the value of personalized dosing compared to standard practice, particularly in health systems where radiotherapy access or resource distribution is constrained [38]. Implementation studies must also address the integration of genomic testing into radiotherapy planning pipelines, including digital platforms capable of merging genomic and dosimetric data in a reproducible and auditable manner.

Together, these steps define a translational pathway that progresses from analytic validation to clinical integration. The ultimate goal is a radiotherapy paradigm in which dose is individualized according to measurable biological properties rather than fixed by tradition, yet implemented with the caution required for interventions that carry substantial risks of long-term sequelae or of reduced tumor control.

Table 2 provides a structured translational roadmap outlining the sequential objectives, requirements and remaining gaps across phases 0 to IV.

**Table 2:** Phase-based roadmap for translational implementation of genomic dose personalization.

Phase	Objectives	Key Requirements	Limitations/Current Gaps
**Phase 0–I (Preclinical & retrospective)**	Derive genomic signatures from SF_2_; link RSI to α in LQ model; perform retrospective validation	Large transcriptomic datasets, clonogenic assays, locked algorithms	Retrospective bias; no dosing personalization tested
**Phase II (Prospective observational)**	Validate analytic reproducibility; assess timing (pre/post-neoadjuvant); evaluate workflow feasibility	Inter-lab concordance studies; standardized pipelines	Does not test dose modification; uncertainty about optimal thresholds
**Phase III (Randomized trials)**	Test RSI/GARD-guided dose de-escalation or escalation against standard of care	Defined clinical thresholds; toxicity safeguards (e.g., RILA); adaptive designs	No threshold consensus; lack of validated post-NACT RSI workflow
**Phase IV (Real-world integration)**	Assess population-level effectiveness, cost-effectiveness, and equity of access	Registries, implementation science, harmonized IVD assays	Variability across institutions; risk of unequal access to genomic testing

### 4.2 Illustrative phase II trial designs for GARD-guided dose modification

To illustrate potential translational pathways, we propose two complementary Phase II single-arm trial concepts testing biomarker-guided dose adaptation in distinct post-neoadjuvant TNBC populations following KEYNOTE-522 standard chemoimmunotherapy [39]. These designs represent feasibility-focused exploratory frameworks rather than definitive protocols.

#### 4.2.1 Proposed design 1: dose escalation in high-risk chemotherapy-resistant TNBC

A potential trial could address TNBC patients with substantial residual disease (RCB-II or RCB-III) following neoadjuvant carboplatin/paclitaxel/anthracycline-based chemotherapy plus pembrolizumab and breast-conserving surgery, a population exhibiting 3-year recurrence-free survival of 75.7% for RCB-II and 26.2% for RCB-III [40]. Retrospective GARD modeling suggests that dose escalation to 53 Gy in 15 fractions achieves GARD≥21, a threshold associated with improved locoregional control [30], in >95% of TNBC patients [15], while the 5 Gy physical increment above standard 48 Gy remains close to the 66 Gy upper limit validated in the Young Boost trial [35], in terms of EQD2 (69.2Gy for an late α/β ratio of 3). Potential eligibility criteria could include women age ≥18 years with RCB-II or RCB-III on surgical pathology, breast-conserving surgery with negative margins, and post-surgical tumor bed specimens containing enough tumor cellularity to ensure reliable RSI calculation; patients with RILA<12% indicating high constitutional radiosensitivity would be excluded [37]. Post-surgical RSI would be calculated and GARD modeled assuming standard 48 Gy/15 fractions integrated boost; patients with GARD<21 would receive dose escalation to 53 Gy/15 fractions delivered as simultaneous integrated boost (tumor bed 3.53 Gy per fraction, whole breast 2.67 Gy per fraction), while those achieving GARD≥21 would receive standard 48 Gy/15 fractions. This single-arm Phase II concept would employ co-primary endpoints of safety (such as composite grade≥3 acute toxicity per CTCAE v5.0 or grade≥2 late fibrosis per RTOG/EORTC at 24 months) and efficacy (3-year actuarial locoregional control), with secondary endpoints including cosmetic outcomes, cardiac dosimetry, and correlation between GARD values and locoregional control. Safety monitoring board review would occur at pre-specified interim analysis with pre-defined stopping rules for excessive toxicity or inadequate locoregional control, requiring multi-institutional collaboration for feasible accrual.

#### 4.2.2 Proposed design 2: boost de-escalation in radiosensitive pathologic complete response TNBC

A complementary trial concept could address TNBC patients achieving pathologic complete response (ypT0/is ypN0, RCB-0) following KEYNOTE-522 neoadjuvant chemoimmunotherapy, a population exhibiting excellent prognosis (95% 3-year recurrence-free survival [40]) yet routinely receiving integrated tumor bed boost despite absence of residual disease. Retrospective modeling on TCGA patients suggests that 80.4% of TNBC breast cancer patients could achieve GARD≥21 without boost [15], indicating potential for adequate biological effectiveness in radiosensitive subsets with opportunity to reduce unnecessary toxicity. Potential eligibility could include women age ≥18 years achieving pathologic complete response after neoadjuvant carboplatin/paclitaxel/anthracycline-based chemotherapy plus pembrolizumab and breast-conserving surgery, with pre-treatment diagnostic core needle biopsy specimens containing enough tumor cellularity for baseline RSI calculation prior to treatment-induced tumor clearance. Pre-treatment RSI would be calculated and GARD modeled assuming whole breast radiotherapy without boost (40.05 Gy in 15 fractions at 2.67 Gy per fraction); patients with GARD≥21 without boost would receive de-escalated treatment consisting of whole breast radiotherapy only with boost deliberately omitted, while those with GARD<21 without boost would be ineligible for de-escalation and receive standard boost per institutional protocols. This single-arm Phase II concept would employ co-primary endpoints of locoregional control (3-year actuarial freedom from ipsilateral breast tumor recurrence) and patient-reported cosmetic outcomes, with secondary endpoints including physician-assessed cosmesis. Safety monitoring board would review safety at pre-specified interim analysis with pre-defined stopping rule for excessive locoregional recurrence, requiring multi-institutional collaboration for adequate sample size and generalizability.

These illustrative Phase II design concepts address therapeutic extremes in post-neoadjuvant TNBC (dose intensification in highest-risk chemotherapy-resistant disease and dose reduction in lowest-risk radiosensitive disease) and could establish feasibility, operational workflows, safety profiles, and preliminary efficacy signals to inform subsequent Phase III randomized trials comparing GARD-guided adaptive dosing versus uniform standard-of-care approaches. Actual trial implementation would require refinement based on regulatory guidance, institutional capabilities, and evolving standards of care.

## 5 limitations of bulk transcriptomics and paths toward refinement

### 5.1 Spatial intratumoral heterogeneity

One of the most significant challenges for genomic personalization of radiotherapy lies in the intrinsic spatial heterogeneity of breast cancer. Even within a single primary tumor, transcriptomic variability is considerable, reflecting clonal diversification, microenvironmental gradients, and differential immune infiltration [41]. These variations directly affect the calculation of RSI and the corresponding estimation of tumor-specific α, ultimately altering the GARD value derived for dose prescription. Importantly, spatial heterogeneity is not limited to the primary tumor. Metastatic deposits in regional lymph nodes may arise from distinct evolutionary trajectories, contain different clonal compositions [42], and harbor divergent radiosensitivity profiles. Consequently, a single biopsy from the primary tumor may not adequately represent the biology of nodal disease, raising the theoretical possibility that optimal dosing for the tumor bed and for nodal basins may differ.

Recent transcriptome deconvolution analyses have further highlighted the sensitivity of RSI to tumor purity. As shown in large-scale breast cancer sequencing cohorts, stromal and immune components substantially influence rank-based gene expression [15], which in turn affects RSI values. Highly inflamed tumors—often dominated by radiosensitive lymphocytes—tend to exhibit artificially low RSI scores when measured from bulk tissue, potentially misleading clinicians toward dose de-escalation. Conversely, poorly infiltrated tumors with substantial stromal content may appear more radioresistant than their malignant compartment truly is. These observations underscore the need for rigorous assessment of tumor cellularity and microenvironmental composition when interpreting RSI-derived metrics for dose selection.

In this context, single-cell RNA sequencing offers a conceptual solution, enabling direct estimation of radiosensitivity at the level of malignant epithelial cells while excluding confounding stromal or immune signals. Such approaches would produce a purer, biologically grounded radiosensitivity estimate. However, widespread implementation remains constrained by the technical difficulty of fresh tissue acquisition, the cost of single-cell workflows, and lengthy processing times incompatible with the clinical radiotherapy pathway [34]. Emerging technologies such as spatial transcriptomics may eventually allow high-resolution mapping of radiosensitivity within tumor sections, offering a compromise between accuracy and feasibility. For now, these methods remain promising research tools rather than practical clinical assays.

### 5.2 Temporal variability and the dynamics of radiosensitivity

Radiosensitivity is not a static trait. Systemic therapy exerts powerful evolutionary pressures on breast tumors, altering both their intrinsic transcriptomic profile and their immune microenvironment [43,44]. In TNBC, tumors with residual disease after neoadjuvant chemoimmunotherapy exhibited a marked increase in RSI compared to baseline biopsies, indicating acquired radioresistance [32]. This phenomenon was accompanied by a reduction in tumor-infiltrating lymphocytes and naïve B cells, suggesting that immune depletion removes a critical component of radiosensitivity captured indirectly by bulk transcriptomic biomarkers.

These findings have profound implications for clinical implementation. If radiosensitivity evolves during systemic therapy, reliance solely on pre-treatment biopsies may substantially underestimate post-treatment resistance, particularly in patients with residual disease. This could lead to underdosing precisely in the group that stands to benefit most from dose escalation. Conversely, patients achieving pathological complete response lack residual tissue for post-treatment profiling, forcing clinicians to depend on pre-treatment RSI despite the uncertain relationship between baseline and final tumor biology.

In cases of preoperative radiotherapy protocols [45], radiosensitivity could be reassessed during therapy, informing mid-treatment dose modulation. This strategy might be particularly appealing for patients with chemorefractory disease [46,47], where persistent viable tumor during neoadjuvant therapy offers an opportunity for repeated biopsies. However, repeated sampling is invasive, logistically complex, and unlikely to be acceptable in routine practice. For tumors treated definitively without surgery, such is the case for frail patients [48], image-guided tumor bed biopsies during radiotherapy could in theory inform adaptive dose intensification, but this approach remains speculative and technically challenging.

Ultimately, the temporal dynamics of radiosensitivity present an unavoidable uncertainty for precision radiotherapy. Prospective trials must determine when and how many timepoints are necessary for optimal prediction, and whether dynamic changes in RSI can be adequately modeled without repeated invasive sampling—potentially using circulating tumor RNA [49] or radiomic surrogates as less invasive biomarkers.

### 5.3 Comparative assessment of radiosensitivity prediction approaches and multiomic approaches

RSI/GARD represents one of several emerging approaches for radiosensitivity assessment, each with distinct advantages and limitations. Radiomics extracts quantitative features from standard imaging (MRI, PET, mammography) to capture spatial heterogeneity in tumor density, perfusion, and metabolism. Radiomic signatures correlate with molecular subtypes and immune infiltration in breast cancer [50] and have shown associations with radiation response pathways in lung cancer [51]. Advantages include non-invasiveness, repeatability for adaptive dosing, and universal applicability without tissue requirements. However, radiomics provides indirect phenotypic surrogates rather than direct molecular measurements, is highly sensitive to acquisition parameters requiring rigorous standardization [52] and lacks prospective validation for radiotherapy personalization in breast cancer.

Functional imaging using hypoxia (18F-FMISO) or proliferation (18F-FLT) PET tracers directly visualizes biological processes governing radiosensitivity [53,54]. While hypoxia-guided dose painting has been explored in head and neck cancer [55], breast cancer applications remain limited by tracer availability, cost, and uncertainty regarding optimal parameters [56]. In addition, the primary breast tumor is surgically excised before radiotherapy, precluding direct functional imaging of malignant tissue. Post-operative imaging captures only the tumor bed microenvironment, not the tumor biology driving radiosensitivity.

Single-cell transcriptomics theoretically offers the highest biological resolution, enabling “pure” tumor cell RSI measurement excluding microenvironmental contamination. However, technical barriers include fresh tissue requirements, high costs ($500–1500 versus $100–300 for bulk sequencing in 2026), extended turnaround times incompatible with radiotherapy scheduling [34] and absence of model for translating clonal heterogeneity into dose prescriptions.

Advances in digital pathology enable extraction of histomorphological features—termed pathomics—from routine H&E slides. These features correlate with immune infiltration [57,58], stromal composition, and proliferative activity [59,60], all of which contribute to radiosensitivity.

Multimodal integration represents the future: transcriptomics quantifies molecular repair programs, radiomics captures spatial heterogeneity, and functional imaging reveals hypoxia *in vivo*. Ultimately, these tools may converge toward the creation of patient-specific digital twins for radiotherapy [61,62]. A complete digital twin would integrate genomic, radiomic, histopathologic, and dosimetric information into a computational model capable of simulating tumor response to different dose regimens. Such models could allow virtual dose escalation trials, enabling oncologists to test hypothetical treatment plans before applying them clinically. While still conceptual, these technologies represent the logical endpoint of radiotherapy personalization and may become achievable as data-rich multimodal cohorts grow and computational methodologies mature.

## 6 translational barriers and implementation pathways

### 6.1 GARD precision and clinical decision-making

Despite rank-normalization inherent to RSI calculation reducing platform dependence, residual technical variability from RNA-seq workflows necessitates standardized protocols for clinical implementation. Key consideration includes tumor cellularity assessment [15], necessitating samples with adequate tumor content or computational deconvolution correction to avoid microenvironmental confounding from stromal and immune components. In addition, comprehensive analytic specifications for optimal GARD calculation (including RNA quality thresholds for FFPE specimens, library preparation protocols, sequencing depth requirements, specific normalization algorithms for gene expression quantification prior to rank-ordering, batch correction strategies, and inter-laboratory reproducibility criteria) remain undefined. Prospective multi-site validation studies establishing these standards do not currently exist, representing a critical translational gap. International consensus development under professional societies to harmonize RNA-seq protocols and define quality control metrics specifically for RSI measurement and GARD calculation is essential before Phase III trial implementation and regulatory qualification as clinical-grade *in vitro* diagnostic assays.

The GARD framework assumes β = 0.05 Gy^-2^ uniformly across patients. From the RSI-derived SF_2_, α is calculated as α = −ln(SF_2_)/2 − 2β, and GARD follows as n×d×(α+β×d). Remarkably, at d = 2 Gy, the β-dependent terms cancel algebraically: GARD simplifies to $n\times d\times \left(-\frac{\ln\;{\text{SF}}_{2}}{2}\right)$, rendering it independent of β. This mathematical invariance protects standard fractionation from β misspecification. However, for hypofractionated schedules (d≠2 Gy), the compensation fails. Expanding GARD yields $n\times d\times \left(-\frac{\ln\;{\text{SF}}_{2}}{2}\right)+n\times \beta \times d\times \left(d-2\right)$, where the residual term n n×β×d×(d−2) introduces β-dependent error that scales with deviation from 2 Gy. For a representative case (SF_2_ = 0.471 [15], 40.05 Gy in 15 fractions at d = 2.67 Gy), assumed β = 0.05 Gy^-2^ gives α = 0.276 Gy^-1^ and GARD = 16.4 Gy. If true β = 0.08 Gy^-2^ (+60% deviation), correct calculation yields α = 0.216 Gy^-1^ and GARD = 17.2 Gy, representing +4.9% error. Thus, substantial β variability (+60%) introduces modest (∼5%) GARD errors in contemporary moderate hypofractionation, demonstrating modest but non-negligible impact in contemporary hypofractionated practice.

A critical consideration for RSI/GARD clinical implementation is SF_2_ prediction uncertainty arising from transcriptomic inference. The original RSI model demonstrated meaningful predictive capacity in independent validation [21] with 5/9 non-leukemia cell lines predicted within ±10% of measured SF_2_ (p = 0.02). Importantly, inter-laboratory variability in clonogenic SF_2_ measurements themselves averages ±17%, with 35% of cell lines showing agreement within ±10% between independent datasets, indicating substantial intrinsic technical variability in the gold-standard assay. Additional factors affecting RSI precision include platform-specific calibration [31], tumor purity effects addressable through computational deconvolution [15], FFPE-related RNA degradation, and batch effects manageable through reference normalization.

To quantify uncertainty propagation into GARD, for standard fractionation (n fractions of d = 2 Gy), $GARD=\left(-\mathrm{Ln}\left({\text{SF}}_{2}\right)\right)\times n$. Differential analysis yields: $\frac{\partial GARD}{\text{GARD}}=\left(-\frac{\partial {\text{SF}}_{2}}{{\text{SF}}_{2}}\times \frac{1}{\left|\ln\left({\text{SF}}_{2}\right)\right|}\right)$. For a median breast cancer SF_2_ ≈ 0.47 [15], |ln(SF_2_)|≈0.75, yielding amplification factor of 1.33 × . Thus, ±10% SF_2_ uncertainty propagates to ±13% GARD uncertainty. representing clinically manageable precision when implementing decision algorithms. GARD-guided prescription should thus incorporate confidence intervals, particularly near decision thresholds (e.g., GARD≈21 [30]). Phase II trials provide opportunity to refine thresholds empirically, with Phase III validation demonstrating improved outcomes versus standard dosing remaining the definitive goal. Refinements such as breast-specific RSI training, standardized RNA-seq protocols with inter-laboratory validation, or computational tumor purity correction will enhance precision toward deterministic prescription frameworks where measurement uncertainty is explicitly incorporated into evidence-based therapeutic algorithms.

### 6.2 Challenges for clinical implementation

The integration of genomic radiosensitivity signatures into breast cancer radiotherapy presents a paradigm shift but also a series of translational challenges that must be addressed rigorously before clinical implementation. Despite robust preclinical foundations and compelling retrospective evidence, current application remains limited by heterogeneity in assay methodology, uncertainty in clinical thresholds, and variation in measurement timing relative to systemic therapy.

A central translational barrier is the absence of standardized workflows for RSI/GARD measurement. Although rank-based gene expression facilitates platform portability, differences in RNA extraction, quality control, normalization, and computational implementation can introduce variability. Without harmonized protocols, inter-laboratory reproducibility remains uncertain and risks undermining confidence in GARD-guided dose decisions. Standardizing these parameters requires consensus-building at an international level, ideally under the control of major professional societies such as ASTRO and ESTRO, and development of a validated *in vitro* diagnostic pipeline with tightly controlled analytic specifications.

Validation represents the second major bottleneck. Existing RSI/GARD thresholds have been derived retrospectively and differ subtly across cohorts. For example, Ahmed et al. proposed GARD ≥21 as a desirable target in TNBC on a first cohort but an a second retrospective analysis on a different cohort in this same study suggest slightly different cutpoint [30]. Such discrepancies may seem small but can have major implications in dose-personalization trials: if the chosen GARD threshold is set too low, dose escalation may lack efficacy; if set too high, escalation may exceed tolerable toxicity. International harmonization is therefore essential to define a robust, clinically meaningful GARD target that can serve as the basis for Phase III trials.

Regulatory considerations further complicate translation. Any biomarker used to direct therapeutic dose modification must meet safety and performance requirements. Regulatory agencies will require evidence that the assay is accurate, reproducible, analytically validated across diverse sample types, and supported by prospective clinical data demonstrating benefit. In addition, the timing of RSI assessment—pre-treatment, post-neoadjuvant, or both—must be formally evaluated and codified into clinical guidelines.

Given these challenges, an international radiotherapy consortium is necessary to coordinate validation efforts, establish analytic standards, harmonize GARD thresholds, and support prospective clinical trials. Such collaboration would ensure that genomic radiotherapy personalization progresses through a structured, safe, and globally consistent translational pathway rather than fragmented, local initiatives.

The clinical translation of genomic radiosensitivity testing raises important questions related to equity and access. Although the cost of targeted RNA-sequencing panels used to derive RSI is modest ($100–300 per sample in 2026), it may still represent a barrier in resource-limited health systems or underserved populations [38]. Without deliberate implementation strategies, GARD-guided personalization could remain confined to well-resourced academic centers, potentially widening existing disparities in cancer outcomes. Demonstrating cost-effectiveness will be essential for broader adoption. Health economic analyses should assess the incremental cost per quality-adjusted life year gained, accounting not only for testing costs but also for potential downstream benefits, such as reduced toxicity through treatment de-escalation or improved local control through escalation in resistant tumors. Such evaluations will be necessary to support reimbursement and integration into routine practice across different healthcare systems. Equity considerations should be incorporated early into implementation pathways. Complementary approaches, such as radiomics-based surrogates of radiosensitivity derived from routinely acquired imaging, may offer a more accessible means of personalization in settings where molecular testing is not readily available. Ultimately, the success of precision radiotherapy will depend on ensuring that its benefits extend beyond specialized centers and are accessible to patients regardless of socioeconomic or geographic constraints.

Overall, while the promise of genomically guided radiotherapy is considerable, realizing its clinical potential requires a coordinated, multidisciplinary, and international effort. Only through rigorous standardization, comprehensive validation, and a robust regulatory framework can RSI and GARD become safe and effective tools for optimizing radiotherapy dosing in breast cancer.

Table 3 summarizes the major analytical, biological and regulatory challenges that currently limit the clinical implementation of RSI/GARD.

**Table 3:** Key translational challenges for clinical implementation of RSI/GARD in breast cancer.

Domain	Challenge	Implications for Clinical Use
**Analytical standardization**	Heterogeneous RNA-seq pipelines, normalization methods, and RSI computation	Limits reproducibility; requires harmonized extraction and QC procedures
**Threshold definition**	No validated clinical GARD or RSI cut-off; retrospective thresholds vary	Risk of under- or over-treatment in prospective trials
**Spatial heterogeneity**	Tumor purity and stromal/immune infiltration distort bulk-RNA RSI estimates	Possibility of misclassifying radiosensitivity; may require deconvolution
**Temporal heterogeneity**	Post-neoadjuvant changes in radiosensitivity not captured by baseline biopsy	Risk of incorrect dose adaptation, particularly in residual TNBC
**Toxicity integration**	Need to incorporate normal-tissue radiosensitivity (e.g., RILA) in dose-escalation strategies	Ensures safety of escalated GARD-guided regimens
**Regulatory framework**	Lack of certified IVD pipelines for RSI/GARD	Hinders multicenter trials and clinical adoption
**Clinical evidence gap**	No prospective data proving that genomic dose personalization improves outcomes	Prevents guideline integration and routine use

## 7 Conclusion

Genomic biomarkers of radiosensitivity, particularly RSI and its integration into the GARD framework, offer a biologically grounded opportunity to individualize radiotherapy dose in breast cancer, addressing the limitations of current uniform prescriptions. Retrospective evidence consistently demonstrates their ability to capture meaningful variability in tumor radioresponse, yet clinical translation remains premature due to the absence of prospective validation, uncertainties regarding biomarker timing, and the influence of spatial and temporal heterogeneity. Standardization of analytic pipelines, international agreement on clinically relevant thresholds, and coordinated methodological frameworks are now essential prerequisites for safely evaluating genomically guided dose modification in future trials.

## CRediT authorship contribution statement

**Pierre Loap:** Writing – original draft, Conceptualization. **Irene Buvat:** Methodology, Supervision, Validation, Writing – review & editing. **Gilles Crehange:** Supervision, Validation, Writing – review & editing. **Youlia Kirova:** Conceptualization, Supervision, Validation.

## Disclosure of interest

The authors declare that they have no competing interests.

## Funding statement

The authors declare that they have no funding and no financial support.

## Declaration of competing interest

The authors declare that they have no known competing financial interests or personal relationships that could have appeared to influence the work reported in this paper.
